# The emergence of a collective sensory response threshold in ant colonies

**DOI:** 10.1073/pnas.2123076119

**Published:** 2022-06-02

**Authors:** Asaf Gal, Daniel J. C. Kronauer

**Affiliations:** ^a^Laboratory of Social Evolution and Behavior, The Rockefeller University, New York, NY 10065;; ^b^Howard Hughes Medical Institute, The Rockefeller University, New York, NY 10065

**Keywords:** collective behavior, distributed computing, social insects, *Ooceraea biroi*, decision making

## Abstract

The sensory response threshold is a fundamental biophysical property of biological systems that underlies many physiological and computational functions, and its systematic study has played a pivotal role in uncovering the principles of biological computation. Here, we show that ant colonies, which perform computational tasks at the group level, have emergent collective sensory response thresholds. Colonies respond collectively to step changes in temperature and evacuate the nest during severe perturbations. This response is characterized by a group-size–dependent threshold, and the underlying dynamics are dominated by social feedback between the ants. Using a binary network model, we demonstrate that a balance between short-range excitatory and long-range inhibitory interactions can explain the emergence of the collective response threshold and its size dependency. Our findings illustrate how simple social dynamics allow insect colonies to integrate information about the external environment and their internal state to produce adaptive collective responses.

## Significance

In this study, we ask how ant colonies integrate information about the external environment with internal state parameters to produce adaptive, system-level responses. First, we show that colonies collectively evacuate the nest when the ground temperature becomes too warm. The threshold temperature for this response is a function of colony size, with larger colonies evacuating the nest at higher temperatures. The underlying dynamics can thus be interpreted as a decision-making process that takes both temperature (external environment) and colony size (internal state) into account. Using mathematical modeling, we show that these dynamics can emerge from a balance between local excitatory and global inhibitory forces acting between the ants. Our findings in ants parallel other complex biological systems like neural circuits.

Sensory thresholding is one of the most fundamental and well-studied computational primitives performed by organisms, where a perceived level of sensory input is compared with an internal variable to generate a binary neural, physiological, or behavioral response. Organisms rely on sensory thresholds to perform critical functions such as responding to threats, detecting prey, and making decisions (1, 2). Sensory thresholds also play important roles as components of more complex tasks such as foraging, navigation, recognition, and communication (3–5). The methodical study of sensory thresholds is a cornerstone of modern neuroscience and has played a key role in connecting cognitive, behavioral, and computational phenomena to neural and biophysical mechanisms (6, 7). At the computational level, thresholds are understood as decisions that optimize costs and benefits associated with responding or not responding in a specific context and are analyzed using signal detection theory (8). Mechanistically, thresholds in biological systems almost always emerge from a balance between two opposing forces: exciting and restoring electrical currents in excitable membranes (9), excitatory and inhibitory neurons in neural networks (10), or interregion connections at the level of the entire brain (11). Similar dynamical phenomena are also abundant in nonneural systems such as the immune system, cellular and microorganismal behavior, or intracellular signaling networks (12–15).

Sensory response thresholds play an important part in the organization of social insect colonies, which process information and perform cognitive-like functions at the group level (16–19). The distribution of individual response thresholds in a colony gives rise to behavioral differentiation and division of labor (20–23), a hallmark of insect societies. Moreover, studies have demonstrated that, in turn, the sensory threshold of colony members can be modulated by their social environment (24, 25), suggesting that threshold dynamics could underlie the adaptation and reconfiguration of collective behavior. However, it is unclear whether sensory thresholding is by itself a computational primitive at the level of the colony and, if so, how it emerges out of the complex interaction network between the individuals in a colony. Colonies of ants and bees collectively perceive and assess their sensory environment and act upon these assessments in a coordinated manner in choice contexts (26–29). Therefore, it is reasonable to hypothesize that ants can coordinate their behavior in response to a sensory input to create a colony-level thresholded response. Mechanistically, the concept of an ant colony as an excitable system governed by exciting and inhibiting interactions between the ants has been proposed to explain activity waves and temporal oscillations, in direct analogy with neural network dynamics (30–33). In principle, similar interactions could also give rise to collective behavioral thresholds.

The analogies and parallels between single-animal and group-level computations have inspired a long line of experimental and theoretical work (29, 31, 34, 35). Yet, our formal understanding of how collective information processing emerges from group dynamics still lags behind our understanding of neural computation. This is mostly due to the lack of convenient experimental paradigms for relating simple, controlled sensory environments to precise measurements of individual and collective responses. Establishing an experimental paradigm for a systematic study of group-level sensory thresholds can therefore contribute to a formal description of emergent collective computation. Such a paradigm should allow one to robustly relate features of controlled sensory environments to the behavioral response of the colony and its members. Here, we study the emergence of a sensory response threshold in colonies of the clonal raider ant *Ooceraea biroi*. The clonal raider ant is an attractive model organism for these kinds of experiments (21, 36–38). It provides unparalleled experimental control over the size and composition of colonies, as well as over the genotype and age of each individual ant in the colony, thus allowing standardization of colony features between experimental replicates. We use a custom setup for controlling the thermal environment of the ants and to measure the behavioral responses of colonies to temperature changes, demonstrating that the collective response is indeed characterized by an emergent sensory response threshold. We then combine experimental manipulations of colony size with mathematical modeling of colony dynamics to investigate the relationship between the collective threshold and the underlying interactions between the individual ants.

## Results and Discussion

### Ants Respond to Increasing Temperature by Evacuating the Nest.

**Fig. 1.**
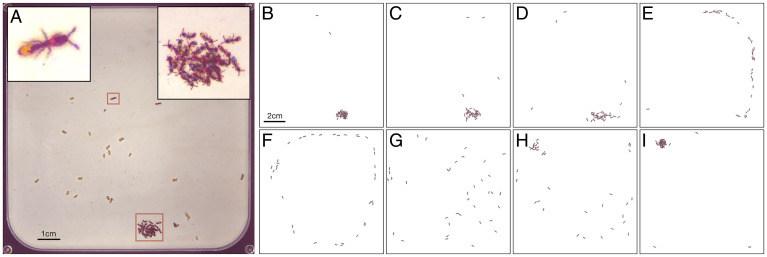
The response of an ant colony to a step temperature perturbation. (*A*) A snapshot from a raw experimental video, showing a colony of 36 ants on a temperature-controlled plaster of Paris arena (*Materials and Methods* and *SI Appendix*, Figs. S1 and S2). Each of the ants is marked with a unique combination of color tags to allow for individual behavioral tracking. The arena is confined by a black metal frame heated to 50 °C (*SI Appendix*, Fig. S2). The ants form a nest (red square at the bottom and *Inset* at top right), with a few scout ants exploring the arena (top red square and *Inset* at top left). The light brown objects in the arena are food items. (*B–I*) Snapshots depicting the typical dynamics of the response of a colony to a strong temperature perturbation. Images are processed by removing the background for visual clarity. (*B*) Baseline state. Before the onset of the perturbation, most ants reside in the nest, with few scout ants exploring the arena. (*C*) Excitement. Following the onset of the perturbation, the ants first respond by increasing their activity level around the nest. (*D*) Evacuation onset. After a delay that lasts up to a few minutes, the ants suddenly begin to leave the nest in a well-defined direction. (*E*) Full evacuation. The colony forms a well-organized evacuation column. (*F*) Stable evacuation. (*G*) Disordered perturbed state. In some cases, especially under high-temperature perturbations, the organized evacuation column breaks, and the colony enters into a high-activity, swarm-like state, where the movements of the ants are only weakly correlated. (*H*) Relaxation. Following the return of the temperature to baseline, the ants slowly relax and begin to reform the nest, possibly in a different location. The relaxation process can take up to 1 h to complete. (*I*) New baseline. The colony has fully returned to its baseline relaxed state.

Controlling the sensory environment of a group of freely behaving animals is challenging. We decided to use thermosensation as the sensory modality, because temperature is a scalar property of the environment, and its sensation is minimally dependent on the position and specific behavior of the individual. To study how ants respond to a temperature increase, we developed a behavioral arena in which the ground temperature is controlled by an array of thermoelectric components (*SI Appendix*, Fig. S1 and *Materials and Methods*). A thin layer of plaster of Paris constitutes the surface of the arena. This setup allows us to rapidly change the arena’s ground temperature (*SI Appendix*, Fig. S2), while maintaining ground moisture level constant (*SI Appendix*, Fig. S3). In all of the following experiments, we placed *O. biroi* colonies of variable sizes in the arena. Unlike most other ants, *O. biroi* is queenless, and all experimental colonies were composed of workers and larvae in a 2:1 ratio. Adult ants were ∼1 mo old, and larvae were 6 to 7 d old. *O. biroi* reproduces asexually and clonally, providing precise experimental control over an individual’s genotype. We standardized genotypes by sourcing all individuals from the same clonal line and stock colony (*Materials and Methods*). At baseline, the set value of the ground temperature controller was 26 °C. When ants are placed in the arena, they quickly create a nest by settling around a brood pile (Fig. 1 *A*, *Inset* and *SI Appendix*, Fig. S4). Typically, a few scouts explore the arena, while most ants remain inside the nest (Fig. 1 *A* and *B*). After a settling period of about 48 h, we studied how colonies respond to temperature changes by subjecting them to a sequence of perturbation events. In each perturbation, the set temperature was abruptly increased to a higher set value for a period of 15 min. Perturbations were spaced by intervals of 2 h to allow the ants to resettle (*SI Appendix*, Fig. S5). When the perturbation temperature was relatively high, the colonies typically responded with a stereotypical coordinated evacuation of their nesting site. Fig. 1 *B–E* and Movie S1 show a representative example of a colony of 36 workers and 18 larvae responding to a 40 °C perturbation. Following the temperature increase, the ants gradually get excited and increase their activity levels (Fig. 1*C*). After some delay, the colony initiates an ordered evacuation in which all ants leave the nest in a column (Fig. 1 *D* and *E* and Movie S1). Because temperature is increased evenly across the entire arena, the ants remain in a high-activity “explorative” state following the initial response. Depending on the perturbation temperature, this state can be highly organized (for relatively low temperatures, Fig. 1*F*) or manifest as a more chaotic and disorganized collective pattern (for high temperatures, Fig. 1*G*). Once the temperature returns to baseline, the ants slowly relax and reform the nest cluster (Fig. 1 *H* and *I*).

### The Colony Response to Temperature Perturbations Is Collective.

**Fig. 2.**
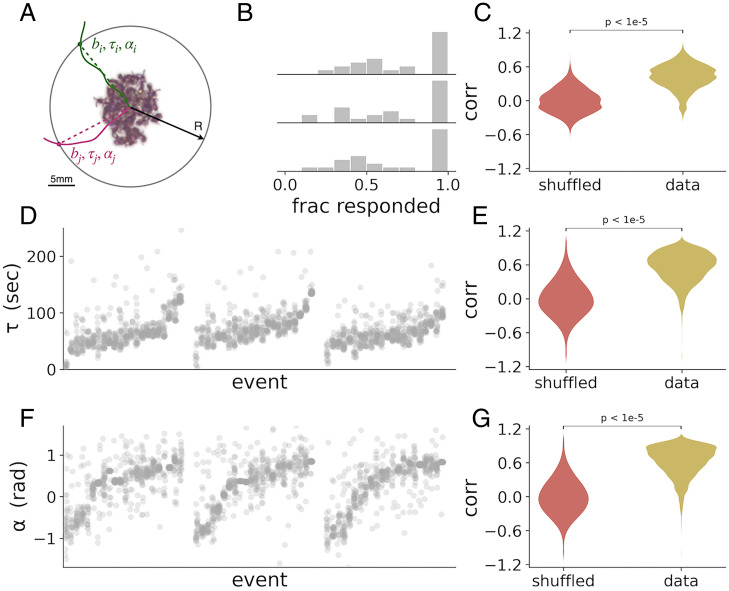
Ants respond collectively to temperature perturbations. (*A*) Measures of individual responses. We define a circle of radius *R* = 15 mm around the location of the nest. A schematic drawing of the trajectories of two ants is depicted in green and pink. For each ant, we record the binary response (b), the response direction (α), and the response latency (τ) as its first crossing of that circle for a duration longer than 30 s, as explained in the text and *Materials and Methods*. (*B*) Histograms of the average binary response across ants. Each histogram is constructed from one colony subjected to a sequence of 24 perturbations of 33 °C. (*C*) Pairwise correlations between the binary responses of ants in *B*, compared to correlations in shuffled responses. Shuffled responses are generated by shuffling the binary responses of each ant to all the perturbations independently of other ants in its colony, therefore eliminating any correlation. The real distribution is composed of 1,890 correlation values, produced from the responses of 108 ants from three colonies. The null distribution is composed of 189,000 correlation values, produced from 100 independent shuffles of the responses. (*D*) Scatter plot depicting the distributions of individual response latencies, from three colonies subjected to a sequence of 24 perturbations of 40 °C. Each column represents the responses of ants from a single colony to one perturbation. The events are sorted first by colony and then by the average individual response latency in each event. (*E*) Pairwise correlations between the response latencies of ants in *D* compared to a null distribution generated in the same way as in *C*. (*F* and *G*) Plots as in *D* and *E*, but for the individual response directions. Note that the response direction measure is cyclic, but because the per-event distribution (one column in *F*) is narrowly distributed around the average colony direction, this does not have a significant effect on the analysis.

Escape, or place-change behavior in response to changes in temperature or other environmental parameters, is a ubiquitous behavior that is often studied in the context of sensory decision making (1, 39–41). Solitary animals make such decisions independently, and the correlations between individuals are generally low, both in the decision itself (whether to leave or not) and in the timing and direction of leaving. In contrast, the response of clonal raider ants to the temperature increase seemed highly coordinated, both in time and in space. To quantify the collectivity in this response, we performed an experiment to measure the coordination and correlation between the responses of individual ants. We perturbed three colonies of 36 ants and 18 larvae with a sequence of 24 temperature perturbations of 15 min duration, each with an amplitude of 33 °C, which does not produce a robust evacuation response. Ants in these colonies were marked with unique combinations of color tags and were individually tracked using a custom software (42). From the tracking results, the nest location before each perturbation was determined (*Materials and Methods*). To identify evacuating ants, we defined a circle with a 15-mm radius around the nest (around twice the typical radius of the nest blob, Fig. 2*A*). The binary response $b_{i}^{k}$ of the i th ant to the k th perturbation was defined as 1 if she exited the nest circle at some point during the time of the perturbation and remained outside for a duration of at least 30 s and as 0 in the case that she did not. Ants that were outside the nest circle at the beginning of the perturbation were treated as missing values. For each of the three colonies, we found that the distribution of average responses across ants for each perturbation is bimodal (Fig. 2*B*), suggesting the decisions to leave the nest are correlated between ants. To test this, we calculated the correlation between the response of each pair of ants in the colony and compared the distribution of these values to a null distribution, constructed by randomly shuffling the individual responses of the ants between perturbation events. These two distributions differ significantly (average pairwise correlation value of 0.424, *P* < 10^−5^; *Materials and Methods* and Fig. 2*C*), showing that the decisions of ants in the colony are indeed highly correlated.

To assess whether the ants are also correlated in the timing and direction of their response, we repeated the experiment with three additional colonies of the same size and composition, using the same protocol. However, this time we subjected the colonies to stronger perturbations of 40 °C, which generally produce a robust collective nest evacuation. We defined the individual response as before and also measured the response delay $\tau_{i}^{k}$ as the time elapsed between the onset of the k th perturbation and the time the i th ant crossed the circle and the response direction $\alpha_{i}^{k}$ as the angle between the point of crossing and the line connecting the nest center and the center of the arena (Fig. 2*A*). Plotting the distributions of individual response latencies across perturbation events, we found that the variability between individual response latencies in the same event is lower than the variability between events (Fig. 2*D*), suggesting that ants are coordinated in the timing of their response. We showed this formally by comparing the distribution of pairwise latency correlations to a null distribution in the same way as before (average pairwise correlation value of 0.558, *P* < 10^−5^; *Materials and Methods* and Fig. 2*E*). We then repeated the same analysis for the response direction, showing that ants coordinate their response also spatially (average pairwise correlation value of 0.628, *P* < 10^−5^; *Materials and Methods* and Fig. 2 *F* and *G*).

### The Collective Response Is Characterized by a Size-Dependent Threshold.

**Fig. 3.**
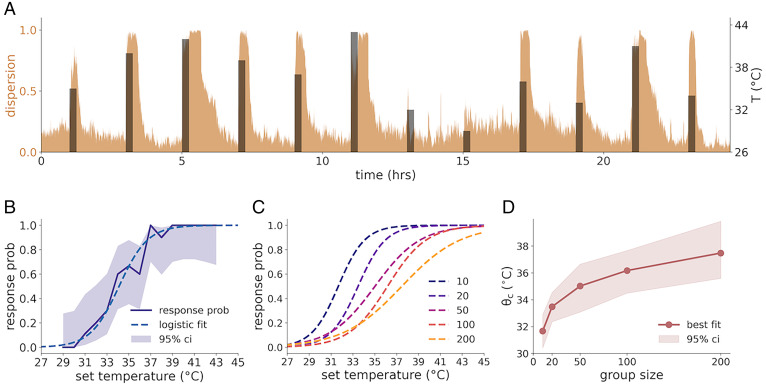
The collective threshold depends on group size. (*A*) A trace from a 24-h-long perturbation protocol using a colony of 36 tagged ants. Perturbations are 15 min long and separated by intervals of 2 h. The set temperature is shown in black. The dispersion of the colony (defined as the fraction of ants outside the nest circle) is shown in brown. The interval allows the colony to relax back to baseline before the next perturbation. (*B*) The probability of a “full response” (defined as at least 90% of the ants being outside the nest at the same time for at least 30 s at some point during the perturbation; solid line) as a function of the perturbation temperature. The shaded band represents the 95% CI of probability (computed using asymptotic normal approximation for binary coefficient estimation). The dashed line represents a logistic regression fit of the response curve. Experimental colonies consisted of 36 tagged ants. (*C*) Fitted logistic regression curves as in *B* for different colony sizes, showing an upward shift in the response curve. (*D*) The collective threshold parameter $\theta_{c},$ estimated by logistic regression, as a function of colony size. The shaded band represents the 95% CI, estimated using the bootstrap method with 1,000 sample repetitions (*Materials and Methods*).

To better understand how the collective response depends on the amplitude of the perturbation, we performed an experiment with 10 colonies of 36 workers and 18 larvae each and subjected each colony to a sequence of perturbations of variable amplitude, ranging from 28 to 45 °C. We defined the collective state of the colony as the fraction of ants outside of the nest in any given frame (Fig. 3*A*). We defined the binary collective response as 1 if a quorum of at least 90% of the colony was outside the nest for at least 30 consecutive seconds at some point during the time of the perturbation and 0 otherwise. For each perturbation temperature, we computed the probability of a collective response across all colonies, resulting in a sigmoidal psychometric-like response curve typical for systems with a noisy threshold response (Fig. 3*B*). Using a logistic regression model (*Materials and Methods*), we estimated the threshold θ to be 34.12 °C for the colonies in this experiment, with a 95% CI of 33.3 to 34.8 °C.

To further investigate this collective threshold, we conducted an experiment with different colony sizes, ranging from 10 to 200 ants. The age, clonal line, and workers to larvae ratio were identical to the experiments described above (*Materials and Methods*). Each colony was subject to the same experimental protocol with varying temperature perturbations. The collective threshold was estimated for each colony size as above (Fig. 3*C*). Plotting the threshold as a function of colony size (Fig. 3*D* and *SI Appendix*, Fig. S6), we found that larger colonies have a significantly higher collective threshold than smaller ones. This effect is robust to variation in the parameters defining the binary collective response (the quorum threshold and duration; *SI Appendix*, Fig. S7).

Such a dependency of an emergent property on group size in a system characterized by many intricate interactions is not surprising from a complex systems science point of view, and an increase in social cohesion as a function of group size has indeed been observed experimentally (43). Nevertheless, it is unexpected in light of previously established paradigms for the study of collective sensing in social insects. These can roughly be divided into two classes that lead to two different predictions regarding group size effects. The first one is “wisdom of the crowd,” in which noisy independent individual estimates of an external signal are pooled to produce a more accurate collective estimate. Under this scenario, the variability in the response is a result of noisy estimation of the external environment by individual ants, and the threshold temperature is an objective quantity that is independent of the group. Accordingly, the prediction would be that larger groups should have higher accuracy (i.e., less variance) in estimating temperature, but the threshold temperature should not change with group size (44–46). A dependency of the threshold on group size would imply that group dynamics integrate information suboptimally and in a biased way (47–49). According to the second paradigm, collective sensing is used to increase the resolution, or sensitivity, to external events such as predator attacks (50, 51). Perturbations of any strength should ideally elicit a response, and the existence of a response threshold is the result of a limited detection capacity. In this case, however, larger groups are predicted to have lower thresholds, because the probability of detecting a weak perturbation increases with the number of individuals in the group. In contrast to these previously documented dynamics, our finding that the collective temperature threshold increases as a function of group size suggests that the response threshold is not an objective quantity to be estimated, but rather a result of a decision-making process that integrates information about the environment with information about the internal state of the colony. It is in fact possible that the optimal collective response threshold differs for colonies of different sizes. For example, if nest evacuation is associated with a relatively higher cost in larger colonies, a threshold that increases with group size might be adaptive.

### The Response Dynamics Are Characterized by Distinct Timescales and Social Feedback.

**Fig. 4.**
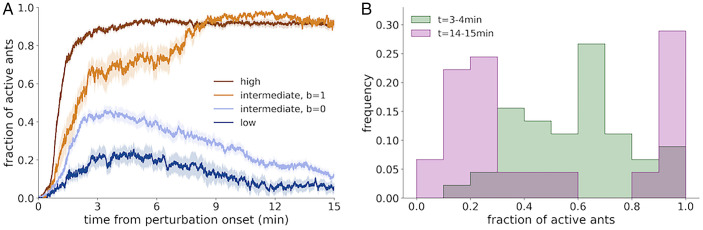
The response dynamics are characterized by distinct timescales and social feedback. (*A*) The evolution in time of the colony state variable (the fraction of active ants) following a temperature change. Single-trial response curves from all experiments with 36 ants were averaged according to temperature and collective response condition. The dark brown curve represents the average response for high-temperature perturbations, for which the response probability was larger than 0.9. Events without a full response (b = 0) were excluded. The light brown curve represents the average response for intermediate-temperature perturbations (response probability between 0.1 and 0.9) in which the colony responded (b = 1). Responses in the same temperature range, but in which the colony did not respond (b = 0), are depicted by the light blue curve. Finally, the dark blue curve represents the average response for low-temperature perturbations [response probability smaller than 0.1; events with positive response (b = 1) were excluded]. (*B*) Histograms showing distributions of single-trial colony activity states for the intermediate-temperature range where the response probability is between 0.1 and 0.9, at two time points along the response curve. The green histogram shows the distribution for the interval between 3 and 4 min following perturbation onset, roughly corresponding to a time window in which the effect of the faster process has been exhausted, while the effect of the slower process is not yet apparent. Each datapoint in the histogram is the median value of a single perturbation event in that segment. The pink histogram shows the distribution for the interval between 14 and 15 min, when both transient dynamics of the response have run their course.

The strong correlation between the responses of individual ants and the existence of a collective response threshold implies that the collective response is coordinated using interactions between the ants, resulting in positive feedback dynamics. To visualize the dynamics underlying the emergence of the collective response, we plotted the average time course of the collective state (the number of active ants outside the nest; *Materials and Methods*) over the perturbation events in the variable-amplitude experiment with colonies of 36 tagged ants (Fig. 4*A*). We divided the perturbation events into three groups, according to the perturbation amplitude: weak perturbations, for temperatures in which the response probability is lower than 0.1; strong perturbations with a response probability greater than 0.9; and intermediate perturbations between those cutoffs. For intermediate perturbations, we separately plotted events in which the colony collective binary response was 1 and 0, respectively (Movies S1 and S2). The time-course plot is indicative of two dominant processes with distinct timescales. The first one is a fast response, with a timescale of 1 to 3 min, in which some ants become excited. This fast response is slower than the timescale of the physical temperature increase (*SI Appendix*, Fig. S2*E*), and the delay might correspond to the internal physiological and neural processing time, as well as the behavioral delay until an ant is considered to have “responded” by our definition. At this timescale, the number of ants responding to midrange temperature perturbations is widely distributed around half of the number of ants in the colony (Fig. 4*B*, green). This is what we would expect if the ants respond independently to perturbations around the typical individual threshold. It is therefore plausible that the colony response during this timescale is dominated by the ants’ intrinsic responses and less by interactions between the ants, which leads to a continuous distribution of the colony state variable (Fig. 4*B*). Following this first stage of the response, a few minutes into the perturbation, a second dynamical process with a characteristic timescale of 5 to 10 min seems to kick in, in which the colony state converges on either a low or a high value (Fig. 4*B*, pink). This convergence indicates the dominance of interaction-driven social feedback during this stage.

### A Binary Network Model Recapitulates the Emergence of a Collective Threshold.

**Fig. 5.**
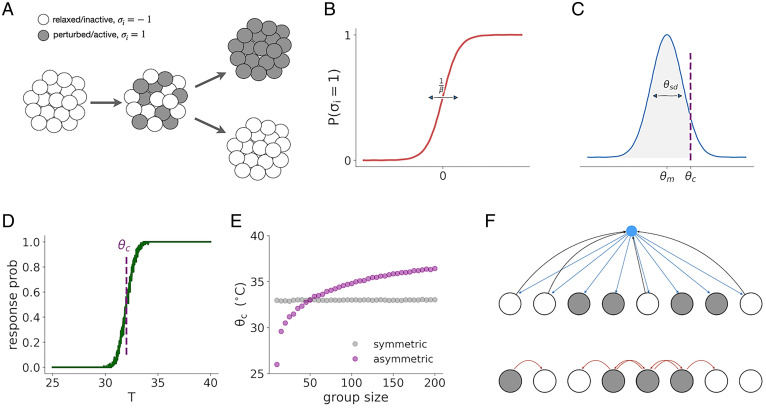
The emergence of the collective threshold can be modeled with two opposing forces. (*A*) An illustration of the model’s two-stage dynamics. In the first stage, the ants respond independently according to their individual response thresholds. As a result, a subset of ants becomes active. In the second stage, the interactions between the ants result in the colony being either fully active or fully inactive. (*B*) The logistic activation function of the individual ant. The ant is either active or inactive in a probabilistic manner depending on an integrated input parameter $h_{i}.$ The β parameter controls the width of the ambiguous response region. (*C*) The individual response thresholds $\theta_{i}$ are sampled from a normal distribution of mean $\theta_{m}$ and width $\theta_{SD}.$ The collective threshold $\theta_{c}$ is the temperature for which the cumulative probability equals $m_{c}.$ (*D*) Simulation of the collective threshold, showing the response probability as a function of temperature, averaged over 100 simulation runs. For each run, a new set of individual thresholds is sampled. See *Materials and Methods* for full details on the simulation parameters. (*E*) The collective threshold as a function of group size for the basic model (gray circles) and the asymmetric model (purple circles). The interaction parameters $J^{p}$ and $J^{r}$ were chosen to have the same collective threshold at N=50 and to approximately replicate the range of thresholds observed in the experiment. (*F*) An illustration of possible ant interaction mechanisms. (*Top*) Global, pheromone-based interaction, in which ants in a given state contribute (black arrows) to the total concentration of pheromone in the environment (blue circle), which is then perceived by all ants (blue arrows). (*Bottom*) Local, contact-based interaction, in which ants in a given state affect only the behavior of nearby ants (red arrows).

To better understand the interactions that might underlie a colony’s collective response, we implemented a simple spin-like binary network model. This type of model is commonly used for complex and collective systems (52–55). We represent each ant by a binary variable $\sigma_{i},$ in which $\sigma_{i}=1$ represents the “perturbed” behavioral state and $\sigma_{i}=-1$ the “relaxed” behavioral state (Fig. 5*A*). The behavioral state of each ant is decided by a logistic activation function (Fig. 5*B*):

[1] $$P\left(\sigma_{i}=1\right)=\frac{1}{1+e^{-\beta h_{i}}}.$$

Here, P represents the steady-state probability of the ant to be in the perturbed state, $h_{i}$ is the integrated input of ant i, and β is a thermal noise-like parameter that measures the determinism of the individual response (with high values leading to a deterministic threshold-like response according to the value of $h_{i}$). In the general case, this integrated input is a combination of the external temperature perceived by the individual ant and the contribution of the social interactions. For simplicity, we take advantage of the apparent separation between the timescales of these two components and assume the dynamics to take place in separate stages (Fig. 5*A*). In the first stage, the ants respond individually and independently to the external temperature, according to the equation:

[2] $$h_{i}=T-\theta_{i}.$$

Here, T is the perturbation temperature and $\theta_{i}$ is the individual threshold of ant i, randomly drawn from a normal distribution with mean $\theta_{m}$ and SD $\theta_{SD}$ (Fig. 5*C*). By the end of this stage, some of the ants will be in the relaxed state and some in the perturbed state (Fig. 5 *A*, *Center*). The state of the colony is characterized by the order parameter m, defined as the fraction of perturbed ants in a colony with N ants:

[3] $$m=\frac{1}{2N}\underset{i}{\sum }\left(\sigma_{i}+1\right).$$

In the second stage, the states of the ants change over time according to the social dynamics:

[4] $$h_{i}(t)=\underset{j}{\sum }J_{ij}\left(t\right)\sigma_{j}(t).$$

Here, $J_{ij}(t)$ is the interaction strength between ant i and ant j at time t. In the most general case, the interaction between a pair of ants will depend on their relative position and on their behavioral states. We further simplify our model by ignoring space and assuming that all ants interact with all other ants all the time. This can be justified by the observation that the collective response dynamics of the ants are much slower than their movement speed in the nest during the “excitement” period that precedes the response (Movies S1 and S2). Under this assumption, we can characterize the interaction by two parameters, representing the exciting effect of perturbed ants and the inhibiting effect of relaxed ants. The input to all the ants is then the same and can be written as:

[5] $$h\left(t\right)=J^{p}N^{p}\left(t\right)-J^{r}N^{r}\left(t\right)=NJ^{p}m\left(t\right)-NJ^{r}\left(1-m(t)\right),$$

where $J^{p}$ and $J^{r}$ are the interaction parameters, and $N^{p}\left(t\right)$ and $N^{r}\left(t\right)$ are the numbers of perturbed and relaxed ants at time t, respectively.

To reproduce the experimental results within the model, we assume β to be high enough for the model to be characterized by two stable fixed points at m=0 and m=1, representing the collective relaxed and perturbed states (*SI Appendix*, Fig. S8 *A–D*), with a separatrix at:

[6] $$m_{c}=\frac{J^{r}}{J^{p}+J^{r}}.$$

This implies that, on average, the value of m at time 0 will decide the final collective state of the colony. This $m_{0}$ is a result of the individual responses of the ants to the temperature increase during the first stage of the dynamics. Therefore, the threshold perturbation temperature $\theta_{c}$ is the temperature at which the average fraction of perturbed ants will equal $m_{c}.$ In the case where the thermal noise is small compared to the individual threshold variability, this threshold satisfies the condition:

[7] $$P\left(\theta_{i}<\theta_{c}\right)=m_{c}.$$

The exact value of this threshold will depend on the distribution of the individual thermal thresholds in the ant population, as well as the ratio between the excitatory and inhibitory interaction strengths (Fig. 5*C* and *SI Appendix*, Fig. S8).

### Group Size Dependency of the Collective Threshold Entails Asymmetric Interactions.

While the model, as defined above, captures the emergence of the collective threshold from the interactions between the ants (Fig. 5*D*), it does not include any group size dependency that can reproduce the experimental observations in Fig. 3 (*SI Appendix*, Fig. S8*L* and gray curve in Fig. 5*E*). Because ants in the experiments had a narrow age range, belonged to the same clonal line, and were sampled randomly from the same stock colony in each experiment (*Materials and Methods*), we can assume that their individual thermal thresholds were also sampled from the same distribution, regardless of colony size. This implies that, under the assumptions of the model, group size dependency can arise only if the excitatory and inhibitory components scale differently with group size N. For example, we can set the inhibitory interaction to scale linearly with N, but let the excitatory interaction be independent of N:

[8] $$h\left(t\right)=J^{p}m\left(t\right)-NJ^{r}\left(1-m(t)\right)$$

[9] $$m_{c}=\frac{NJ^{r}}{J^{p}+NJ^{r}}.$$

Using these definitions, the threshold value will sublinearly increase with the size of the colony (purple curve in Fig. 5*E*).

This choice is not arbitrary, because different types of interactions should produce different scaling with N. Clonal raider ants are blind and mostly communicate via pheromones and tactile interactions. For a volatile pheromone, the concentration in the air surrounding ants aggregated in close proximity in the nest should scale with the number of ants that emit the pheromone, which equals the fraction of pheromone-emitting ants multiplied by N. This pheromone concentration is then perceived by all ants in the colony (Fig. 5 *F*, *Top*). The exact form of the scaling will depend on the chemical properties of the pheromone, the physical properties of the environment, and the concentration/response curve of the ants themselves. On the other hand, physical interactions between pairs of ants will depend on the rate of encounters. Physical interaction is a common excitatory mechanism in ants, particularly in scenarios of recruitment (56, 57). When the colony is dense, the encounter rate per ant saturates, meaning an ant is always in contact with other ants at the maximum capacity. This implies that the total excitatory force an ant feels is dependent on the fraction of active ants in the colony and does not scale with the size of the colony (Fig. 5 *F*, *Bottom*). Of course, the distinction between the two mechanisms does not have to be clear cut and can be quantified with a scaling parameter α:

[10] $$h\left(t\right)=N^{\alpha_{p}}J^{p}m\left(t\right)-N^{\alpha_{r}}J^{r}\left(1-m(t)\right).$$

The value of α represents a gradual transition between local, nearest-neighbor interactions and global, all-to-all interactions. An increase of collective threshold with group size will then emerge for any $\alpha_{r}>\alpha_{p}$ (*SI Appendix*, Fig. S9).

So far, we have considered interactions that are state dependent, that is, interactions in which ants signal their state to other ants. However, some interactions within the colony can be regarded as state independent or to vary slower than the typical timescale of the behavioral response. For example, weakly volatile “aggregation” pheromones could mark the nest site. The strength of this nest odor will then scale with the number of ants in the nest but will not change because of ants leaving the nest momentarily. The existence of such a pheromone is supported by the tendency of the ants to settle back at their original nest location following perturbations (*SI Appendix*, Fig. S10). Such an aggregation signal would act as a constant pulling force that balances the excitation within the colony. In our model, we can account for such an interaction by removing the state dependency from the inhibitory term and write Eq. 10 as:

[11] $$h\left(t\right)=N^{\alpha_{p}}J^{p}m\left(t\right)-N^{\alpha_{r}}J^{r}.$$

Because the scaling of the inhibitory interaction strength is the same as in the initial version of the model, we again get an increase of the threshold with group size (*SI Appendix*, Fig. S9 *D* and *E*). However, the shape of the increase and the predictions of the model for larger group sizes differ (*SI Appendix*, Fig. S9*F*).

## Conclusion

Our results provide a simple, tractable example of a collective perception–action loop, where social dynamics are used to integrate the sensory perception of individual ants and to produce a coherent collective response. We show that under borderline conditions, individual ants suppress their own assessment or perception of sensory information about the external environment in favor of a collective decision. Moreover, the social dynamics enable the colony to integrate information not only about the external environment, but also about the state of the colony itself (its size in this case). The collective outcome is therefore more than a mere average of the “opinions” of the individual ants.

Our modeling results also highlight the importance of heterogeneity in social groups. This group-level property is thought to contribute to a group’s ability to adapt to changing conditions in systems ranging from insect colonies to human societies (58–60). For example, in honey bee and bumblebee colonies, the variability of individual thresholds for fanning, a behavior that helps circulate the air, is hypothesized to contribute to the overall performance of collective thermoregulation (24, 61, 62). In our model, heterogeneity is expressed as the distribution of individual response thresholds in a colony. The collective threshold, which is an emergent property of the group, can then vary within the range of that distribution depending on the context, which in our case is the size of the colony. In other words, the variability between individuals is what enables the adaptation of the collective property.

The collective sensory threshold seems to emerge from a balance between two opposing forces. As in other biological systems, these forces do not have to map to a single biological mechanism, but could rather represent a combination of various processes with a similar functional effect. For example, excitation and inhibition in a neural network arise from many different types of neurons and neurotransmitters whose effects differ in aspects such as timescale, spatial distribution, and plasticity. Likewise, transmembrane currents are the product of many types of ion channels, each modulating the membrane response in a different way. This mechanistic complexity underlies both the robustness of biological systems and their flexibility to adapt their responses to various conditions on multiple timescales (63–68). Similarly, the excitatory and inhibitory forces at play in an ant colony are likely composed of various chemical, physical, and possibly other types of interactions. The isolation of individual mechanisms and the understanding of their precise functional role in the collective dynamics will require further experiments. However, mesoscopic models such as the one employed here can provide a formal understanding of the principles of emergent collective computation even without detailed knowledge of the underlying mechanisms.

## Materials and Methods

### Experimental Setup.

The 10 × 10-cm behavioral arena consists of a 3-mm-thick plaster of Paris layer on top of a temperature-controlled metal platform. The platform is divided into four zones. The temperature of each zone is measured by an embedded thermistor (Omega 44304) and controlled by a thermoelectric cooling (TEC) device (CP60; CUI Inc.). The plaster arena is surrounded by a metal frame heated to a high temperature (between 45 and 50 °C) to confine the ants. The arena and the frame are installed inside a box in which the air temperature and the relative humidity (RH) are continuously monitored. *SI Appendix*, Fig. S1 gives full details and a schematic of the experimental setup.

### Temperature Control.

The temperature in each of the arena zones is regulated by an Arduino-implemented proportional–integral–derivative (PID) controller. An additional PID is used to control the temperature of the heated frame. The spatial integrity of the surface temperature is verified by an infrared thermal camera (FLIR Lepton 3.0). Details of implementation and verification are given in *SI Appendix*, Fig. S2. All temperature values reported are the set temperature of the PID, while actual ground temperatures can be inferred from the calibration curve (*SI Appendix*, Fig. S2*B*).

### Moisture Control.

*O. biroi* ants are sensitive to moisture and humidity, and a drop in these parameters will affect their behavior. Without additional measures, heating the plaster floor during temperature perturbations would lead to desiccation. We therefore developed a method to maintain a stable moisture level of the plaster during the experiments. We found that the color of the plaster is a robust and reliable measure for moisture level (the plaster gets lighter/brighter as it dries). We therefore defined the “moisture index” as the 90th percentile of the pixel brightness value distribution in the arena (*SI Appendix*, Fig. S3 *A* and *B*). This definition is robust to the presence of ants and accumulation of trash in the arena during the experiment, because both contribute to the low-brightness tail of the distribution. As the baseline color of the arena depends on batch effects of the plaster, as well as on the exact tuning of the camera and illumination, the set point was manually determined before each experiment. At the start of each experiment, we calculated the pixel brightness distribution and the moisture index for the arena when dry and when completely saturated with water (*SI Appendix*, Fig. S3*B*). We then defined the threshold moisture index $M_{c}$ as:

[12] $$M_{c}=M_{s}+0.1\cdot \left(M_{s}-M_{d}\right),$$

where $M_{s}$ and $M_{d}$ are the respective indexes under saturated and dry conditions. During an experiment, the moisture index was calculated every second (*SI Appendix*, Fig. S3*C*), and whenever it rose above threshold, the waterflow into the embedded water delivery tubes (*SI Appendix*, Fig. S1) was opened for a short (0.5 s) duration. *SI Appendix*, Fig. S3*C* depicts an example time course of the moisture index when the control is enabled (left of the dashed line) and when it is disabled, and the plaster is allowed to dry (right of the dashed line).

### Experimental Design.

Experimental colonies were composed of age-matched, one-cycle-old workers and 6- to 7-d-old larvae in a 2:1 workers to larvae ratio. All ants were derived from the same stock colony (STC6), which belongs to *O. biroi* clonal line B (69). For the threshold experiment, two datasets were collected: one with individually tagged ants in 10 colonies of 36 adults and 18 larvae each and one with untagged ants and variable colony sizes of 10, 20, 50, 100, and 200 ants, as well as 5, 10, 25, 50, and 100 larvae, respectively. For the latter dataset, each group size was represented by 3 replicate colonies (15 colonies total). Colonies were assayed sequentially, one per week, between October 2018 and August 2019. The experiments did not have any specific order. For the collectivity measurement experiment, four colonies of 36 tagged ants and 18 larvae were used.

### Colony Preparation.

Five-day-old workers were separated every other week from an *O. biroi* stock colony and split into two experimental colonies. These ants laid eggs ca. 10 d later. In the first colony, we waited for larvae to hatch and began experiments when larvae were 6 to 7 d old. In the second colony, we removed the first batch of eggs after 3 d. This resets the colony’s reproductive cycle and creates a 1-wk developmental lag between the first and the second colony, allowing us to run experiments continuously. Right before experiments began, colonies were adjusted to the number of ants and larvae required for the experiment of that week and transferred into the experimental setup.

### Color Tagging.

For experiments with individually tagged ants, 12-d-old ants were marked with color dots on the thorax and gaster using oil-paint markers (UniPaint markers PX-20 and PX-21) (21, 70). Colonies for these experiments contained 36 ants, marked with all unique combinations of blue, green, orange, pink, purple/red, and yellow.

### Experimental Protocol.

After experimental colonies had been transferred to the experimental setup, they were allowed to settle for 48 h. During that time, the set temperature of the arena was 26 °C and the ants were fed fire ant (*Solenopsis invicta*) pupae. Approximately 6 h after the last feeding event we began the perturbation protocol, and we did not feed the ants for the rest of the experiment. Every 2 h, the set temperature of the arena was increased to the perturbation value for 15 min and then lowered back to the baseline temperature. The 2-h intervals allowed colonies to resettle and return to baseline activity levels and produce stationary response statistics throughout the duration of the experiment (*SI Appendix*, Fig. S5). For threshold measurements, the sequence of perturbations was a random permutation of the values between 29 and 43 °C. The sequence was presented in the same order for all colonies in the experiment. For the collectivity experiment, colonies were subjected to a sequence of identical perturbations of either 40 or 33 °C.

### Video Recording and Tracking.

Videos were recorded at 10 frames per second using a FLIR Flea3 camera (image size 2,500 × 2,500 pixels). The videos were analyzed using anTraX, a software package for video tracking of color-tagged ants (42). For experiments with tagged ants, the software outputs an estimated location for each ant in the colony in each frame. For experiments with untagged ants, the software outputs a list of segmented blobs containing ants, together with their respective centroid coordinates and area.

### Defining the Location and Size of the Nest.

Most of the behavioral measures we use in our analysis depend on the location of the nest. Because we do not use a physical nest structure, and because the nest location is dynamic and changes during an experiment, we estimate the location and size of the nest from the tracking data. Typically, 50 to 90% of the ants in the colony will reside in the nest at any given time. Therefore, the median location of all the ants will give an accurate estimate of the nest centroid. To be consistent between tagged and untagged experiments, we do not use the individual location data for estimating the nest location. Rather, we use the relative area of each blob as a proxy for the number of ants in that blob, and the equation to determine the nest location takes the following form:

[13] $$x_{\mathrm{nest}}^{\text{t}}=\mathrm{wmed}\left({\left\{a_{i}^{t},x_{i}^{t}\right\}}_{i=1}^{K}\right).$$

In Eq. **13**, $a_{i}^{t},x_{i}^{t}$ are the area and the coordinates of the i th blob at time t, K is the number of blobs in the frame, and *wmed* is the weighted median function. We further use a median filter with duration k=10 min  to suppress fluctuations in this measure:

[14] $$x_{\mathrm{nest}}^{\text{t}}=\mathrm{med}\left({\left\{\mathrm{wmed}\left({\left\{a_{i}^{t},x_{i}^{t^{\prime }}\right\}}_{i=1}^{K}\right)\right\}}_{t^{\prime }=t-k}^{t}\right).$$

We chose the nest to have an effective radius $R_{\mathrm{nest}}$ equal to the major semiaxis of the nest blob. The nest blob, as segmented by the tracking algorithm, represents the tight aggregation of ants in the center of the nest. As in the case of the nest location, we use median filtering to smooth fluctuations. For the analysis of individual and collective responses to perturbations, we use the nest location and radius at the onset time of the perturbation.

### Individual Response Measures and Collectivity.

We define an individual ant as having responded to the perturbation when she has first exited a circle of radius 15 mm around the nest for a duration of at least 30 consecutive seconds (Fig. 2*A*). This radius was chosen so that ants that are excited or active near the nest will be mostly inside the circle, while ants evacuating the nest will exit the circle. We then characterize the response of each ant for a given perturbation using three measures. The binary response $b_{i}^{k}$ is set to 1 if the *i* th ant leaves the circle during the duration of the *k* th perturbation for a consecutive period of at least 30 s. The response delay $\tau_{i}^{k}$ is defined as the delay between the onset of the perturbation and the time the ant first crossed the circle for such a period, and the response direction $\alpha_{i}^{k}$ is defined as the angle of the point of crossing from the line connecting the nest’s centroid to the center of the arena. We then calculate the correlation between the measures for each pair of ants in the same colony. In the case that an ant was outside the circle at the onset of the perturbation, we treat its response to that particular perturbation event as a missing value. Statistical significance (*P* value) was estimated nonparametrically by randomly permuting the responses of each ant to all the perturbations in the experiment and calculating the distribution of pairwise correlation coefficients for this shuffled dataset. For each of the shuffled datasets we then calculated the mean pairwise correlation as in the real dataset. We repeated this process *n* = 100,000 times and generated a null distribution, defining the *P* value as the proportion of the null distribution that exceeds the experimental value. For none of the three response measures did the null distribution yield any value that exceeded the experimental value, entailing *P* < 10^−5^.

### Measuring the Collective Threshold.

We define the colony activity variable $f_{\mathrm{out}}^{t}$ as the fraction of ants in the colony that are outside the nest at time t. As before, to be consistent across experiments with tagged and untagged ants, we do not use the individual location data, but rather an estimate based on blob sizes:

[15] $$f_{\mathrm{out}}^{t}=\frac{\sum_{i|d_{i}<R_{\mathrm{nest}}}a_{i}^{t}}{\sum_{i}a_{i}^{t}}.$$

Here, $d_{i}$ is the distance of the *i* th blob from the nest, and $R_{\mathrm{nest}}$ is the effective nest radius at the beginning of the perturbation, as defined above. Note that in the context of the collective state, we use a smaller circle around the nest than in the case of the individual response, to better capture the activity of ants around the nest and not only ants evacuating the nest.

We define the colony as having responded if $f_{\mathrm{out}}^{t}>0.9$ for a period of at least 30 consecutive seconds during a perturbation. To estimate the response threshold, the responses of 10 colonies in the experiment with tagged ants were pooled. Events in which the ants were not well settled at the beginning of the perturbation were excluded from the analysis. For inclusion, we required the average number of active ants in the 15 min preceding the onset of the perturbation to be lower than twice the median value, calculated across all the events in the experiment. This resulted in the exclusion of 8 of the 150 events. The threshold was estimated by fitting a logistic regression model to the collective binary response variable. CIs for the threshold were estimated using the case resample bootstrap method with 1,000 replicates.

For the group size experiment, we repeated the analysis above, resulting in a threshold parameter and CI for each group size (Fig. 3*D*). The effect of colony size was estimated using a logistic regression model with group size and temperature as the independent variables (*SI Appendix*, Fig. S7).

### Simulation of the Mathematical Model.

The binary network model was simulated using the asynchronous update approach. In each simulation step a randomly selected ant updates its state. In each simulation run, the ants were initialized at the inactive state $\sigma_{i}=-1.$ Each run lasted 20 full update cycles; an update cycle is defined as N simulation steps, where N is the number of ants in the simulated colony. The full simulation parameters are given in the legends of *SI Appendix*, Figs. S8 and S9.

All parameter sweeps were performed on the Rockefeller University high-performance computing cluster.

## Supplementary Material

Supplementary File

Supplementary File

Supplementary File

## Data Availability

All behavioral data, simulation code, and data analysis scripts used in this paper have been deposited in Zenodo, https://doi.org/10.5281/zenodo.6569620 (71).
