# Supplementary material for: The emergence of a collective sensory response threshold in ant colonies
Source: Proc Natl Acad Sci U S A. 2022 Jun 2;119(23):e2123076119. doi: 10.1073/pnas.2123076119 (PMC9191679; doi:10.1073/pnas.2123076119)
Supplement: Supplementary File [file pnas.2123076119.sapp.pdf]

Supplementary Information for
The emergence of a collective sensory response threshold in ant colonies.

Asaf Gal & Daniel J. C. Kronauer

Emails: agal@rockefeller.edu; dkronauer@rockefeller.edu

This PDF file includes:

Figures S1 to S10
Legends for Movies S1 & S2

Other supplementary materials for this manuscript include the following:

Movies S1 & S2

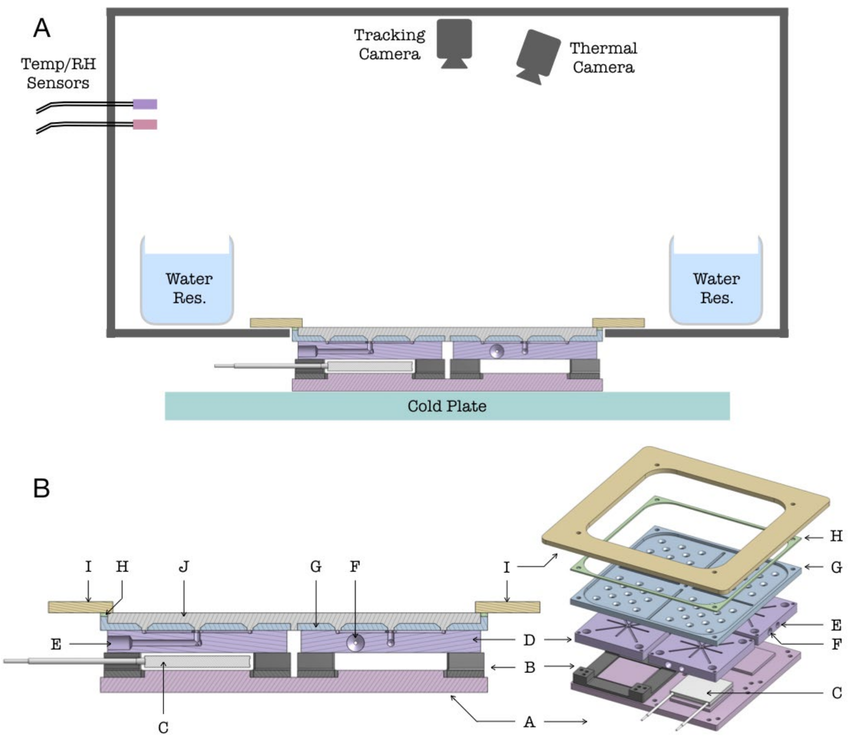

Figure S1: Experimental setup. (**A**) Schematic of the experimental setup (not to scale). The plaster arena and the temperature control platform are situated inside a closed box, together with a water reservoir to reduce the rate of humidity loss from the plaster. The ambient temperature and relative humidity are continuously monitored. A tracking camera records movies of the ants' behavior, and a thermal camera monitors the temperature on the arena's surface. The temperature control platform is placed on a cold plate to sink the excessive heat generated by the thermoelectric coolers (TECs). The custom electronic controls used to drive the TECs and the water flow, and the computer with a custom software used to collect and record data (movies and synchronized temperature and humidity measurements), are not shown. (**B**) Detailed to-scale schematics of the temperature control platform. A: Bottom metal base layer. B: Thermally insulating spacer and support, one for each TEC device (not all are shown). C: TEC device, one for each zone. Thermally conductive silicone pads that attach the device to the metal parts below and above are not shown. D: Middle metal layer, which contains the water tubing and the zone's thermistor. This layer is made of 4 pieces separated from each other to enable individual control of each zone. E: Tubing for water flow, machined into the middle metal piece. Each metal piece contains one inlet which opens up at the surface to distribute the water to 13 outlets into the plaster. F: Hole for thermistor. G: Top metal plate, in which the plaster arena is cast. The top plate is divided into 4 separate zones, which are connected with minimally sized bridges to enable separate temperature control of the four zones (this feature of the setup was not used in the current study; all zones were set to the same temperature in all experiments). The top metal plate is attached to the middle metal pieces (D) using thermally conductive silicone pads (not shown). H: Thermal insulation layer between heated barrier and top metal layer. I: Heated metal barrier (3mm thick aluminum plate). Not shown are the attached thermistor and heating resistors used to control the barrier's temperature. J: Plaster arena, cast onto the top metal plate (G).

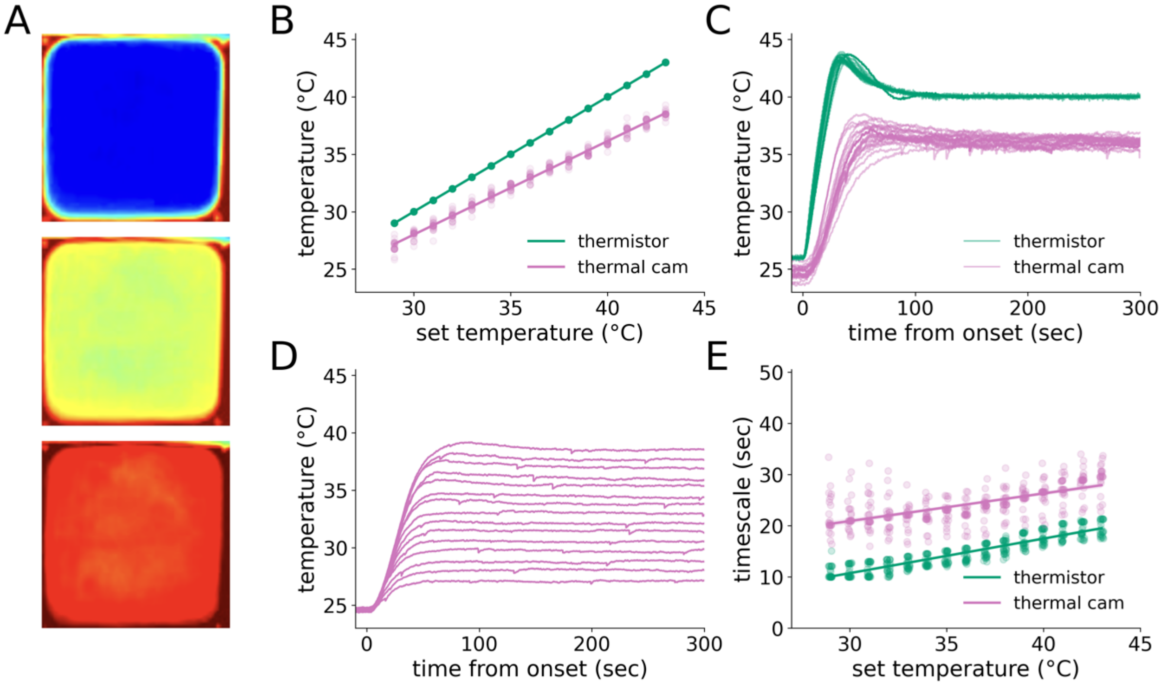

Figure S2: Temperature control. (**A**) Snapshots from the thermal camera during an experiment, for set temperatures of 26°C, 34°C and 40°C (top to bottom). The temperature-controlled arena is surrounded by a high-temperature heat fence that is used to confine the ants to the arena even under the maximum perturbation. (**B**) Calibration curve showing the actual temperature vs. the set temperature. The plot shows the thermistor measurement (green), and the temperature measured by the thermal camera (pink). Each scatter point is data from one perturbation, and the plot shows pooled data from all experiments analyzed in this paper. Both the thermistor and thermal camera measurements are time averages of the measurements between 5 minutes and 15 minutes after the onset of the perturbation. The thermistor measurements are averaged over the 4 thermistors of the arena, and the thermal camera measurements are averaged over the pixels of the arena region of interest. Because the thermistor measurements provide feedback to the TEC controllers, they display 1:1 correspondence with the set temperature with minimal variability. The thermal camera curve is linear with a slope of slightly less than 1, implying a temperature drop across the layer of plaster. The variability in the y-axis is mostly due to the instability in the thermal camera measurements between experiments, and not because of actual variability in temperature across experiments. Because of the low reliability of the thermal camera absolute measurements, we do not use them for control, but only for analysis of the spatiotemporal dynamics of the arena's temperature. (**C**) Comparison of the temperature dynamics as measured by the embedded thermistor (used for temperature control, green) and the thermal camera (pink), for a perturbation setpoint of 40°C. While the temperature underneath the plaster climbs fast and displays a significant overshoot as a result of the PI control algorithm, the ground temperature changes slower and the overshoot is dampened by the mass of the plaster layer. (**D**) Temperature traces from the thermal camera, averaged across perturbations with the same set temperature, for set temperatures between 29°C and 43°C. (**E**) The rising timescale of the single-perturbation temperature trace, defined as the latency from the setpoint change time to the half rise time of the temperature, for the thermistor and the thermal camera. The timescale was estimated by fitting a single exponential to the trace between 10 seconds and 60 seconds following the onset of the perturbation. There is a slight increase in timescale for higher temperatures. This increase is expected because of the use of a PI controller to set the TEC current. The range of variation in the heating timescale is much lower than the typical response time of the colony (Figure 4A).

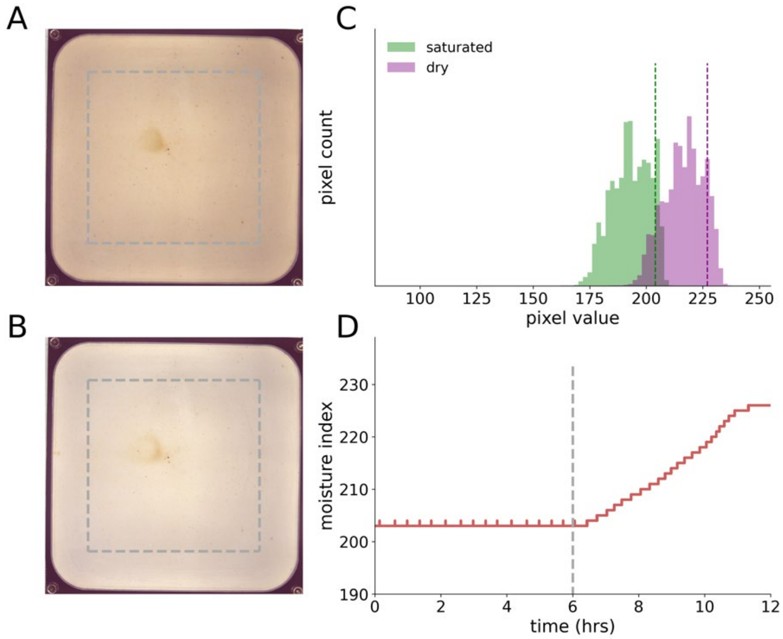

Figure S3: Moisture control. (**A-B**) To calculate the moisture index, the distribution of pixel brightness levels in the arena, excluding margins of 1cm width (dashed grey line), is calculated. Depicted are examples of a fully saturated (**A**) and a completely dry (**B**) frame. (**C**) At the start of each experiment, the pixel brightness distribution is calculated for the arena when dry (purple), and when completely saturated with water (green). A moisture index is then calculated from each distribution as the 90th percentile of the brightest pixels (dashed lines). (**D**) An example time course of the moisture index when moisture control is enabled (left of the dashed line), and when it is disabled, and the plaster is allowed to dry (right of the dashed line). The small spikes in the left part of the curve represent times at which the index increases above threshold and is immediately compensated by the controller.

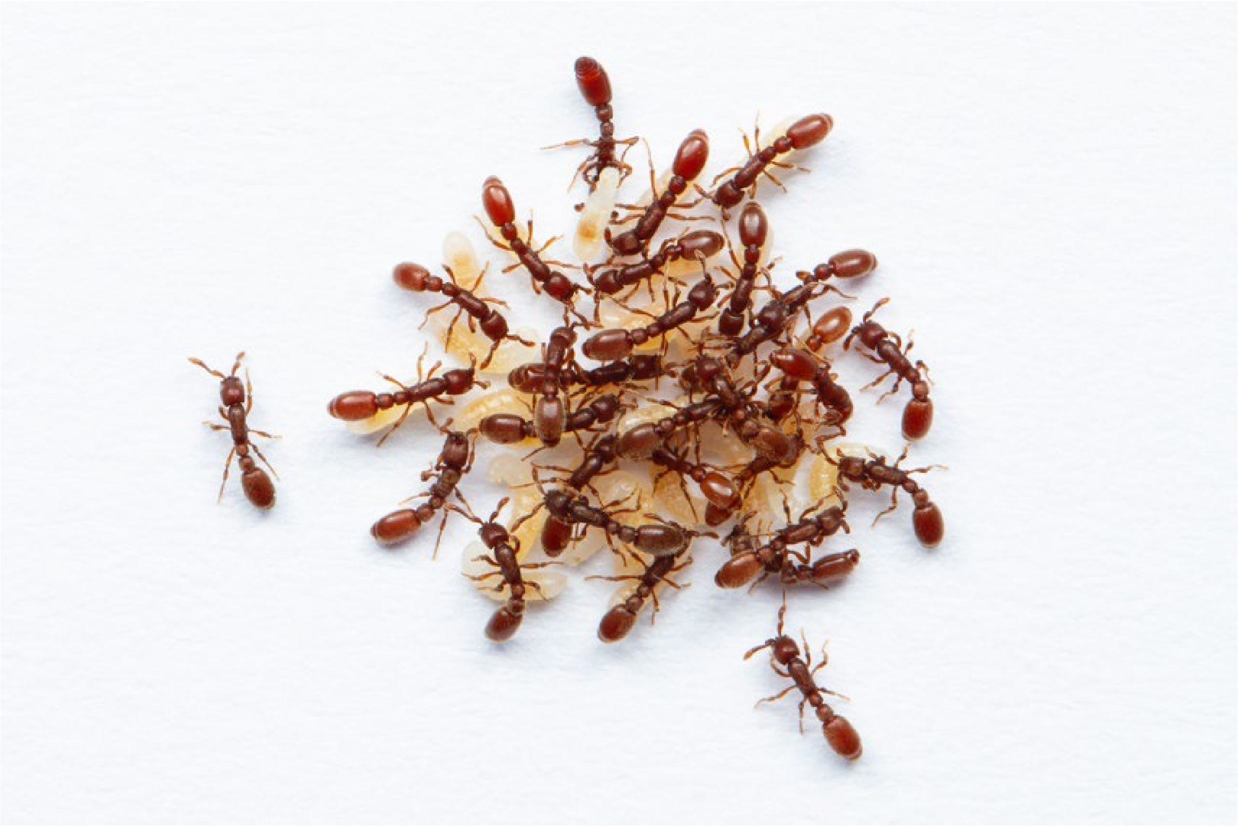

Figure S4: High-resolution image of an *O. biroi* nest blob. During baseline behavior, *O. biroi* colonies assemble into a nest aggregate that contains the brood, and in which most of the ants reside most of the time. This picture was taken with a DSLR camera outside of the tracking system used for this study.

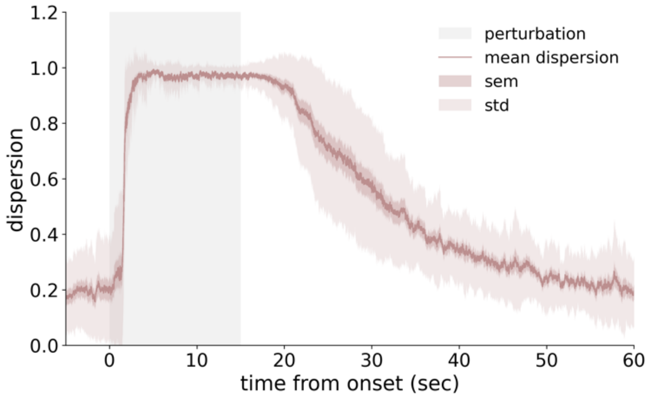

Figure S5: Colony relaxation following a perturbation. Colonies are perturbed with a temperature increase every two hours, for a duration of 15 minutes each time. These parameters were chosen so that colonies will relax back to their baseline activity levels before the next perturbation. Depicted are the mean (line), SEM (dark shaded area) and STD (light shaded area) of activity levels in a single colony, calculated across 24 consecutive perturbation events. The curve shows that it takes around 1 hour for the colony to return to baseline activity.

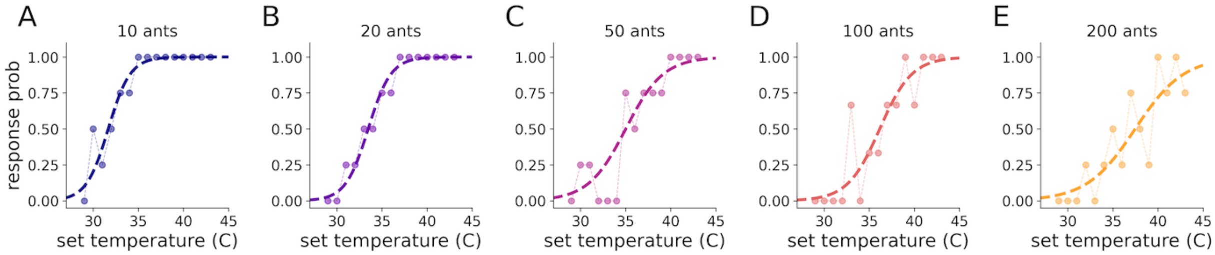

Figure S6: Logistic regression on group size data. (**A**-**E**) Each panel depicts the fraction of perturbations that elicited a collective response for each temperature level for a specific colony size, along with the fitted logistic curve. The curves are identical to those in Figure 3C. The response threshold increases with colony size.

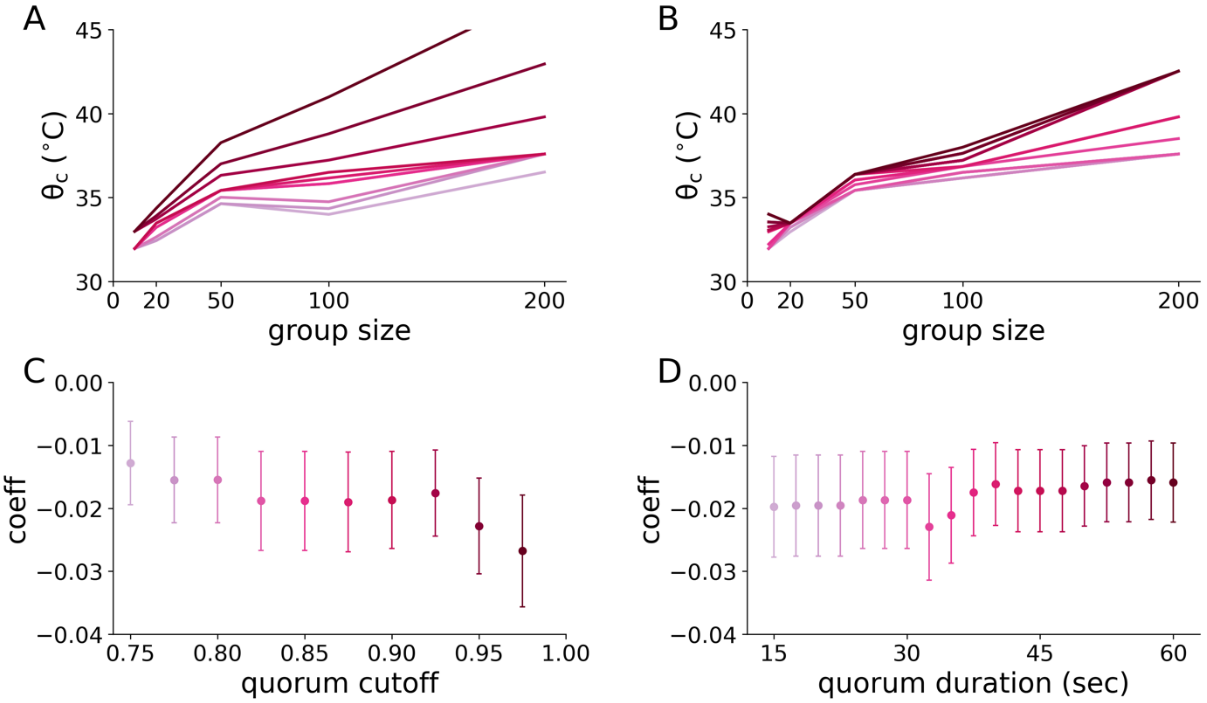

Figure S7: The effect of group size is robust to changes in analysis parameters. (**A**) The threshold as a function of group size for different values of the quorum cutoff parameter, from 0.75 (light) to 0.975 (dark). The threshold data were generated as in Figure 3D. (**B**) The threshold as a function of group size for different values of the quorum duration parameter, from 15 seconds (light) to 60 seconds (dark). The threshold data were generated as in Figure 3D. (**C-D**) The effect of group size on response probability is significant for all tested parameter combinations. The figure depicts the coefficient of the group size parameter in the logistic regression model, together with its 95% confidence interval, for each parameter value tested. Negative coefficients imply a decrease in response probability (i.e., an increase in threshold) with group size.

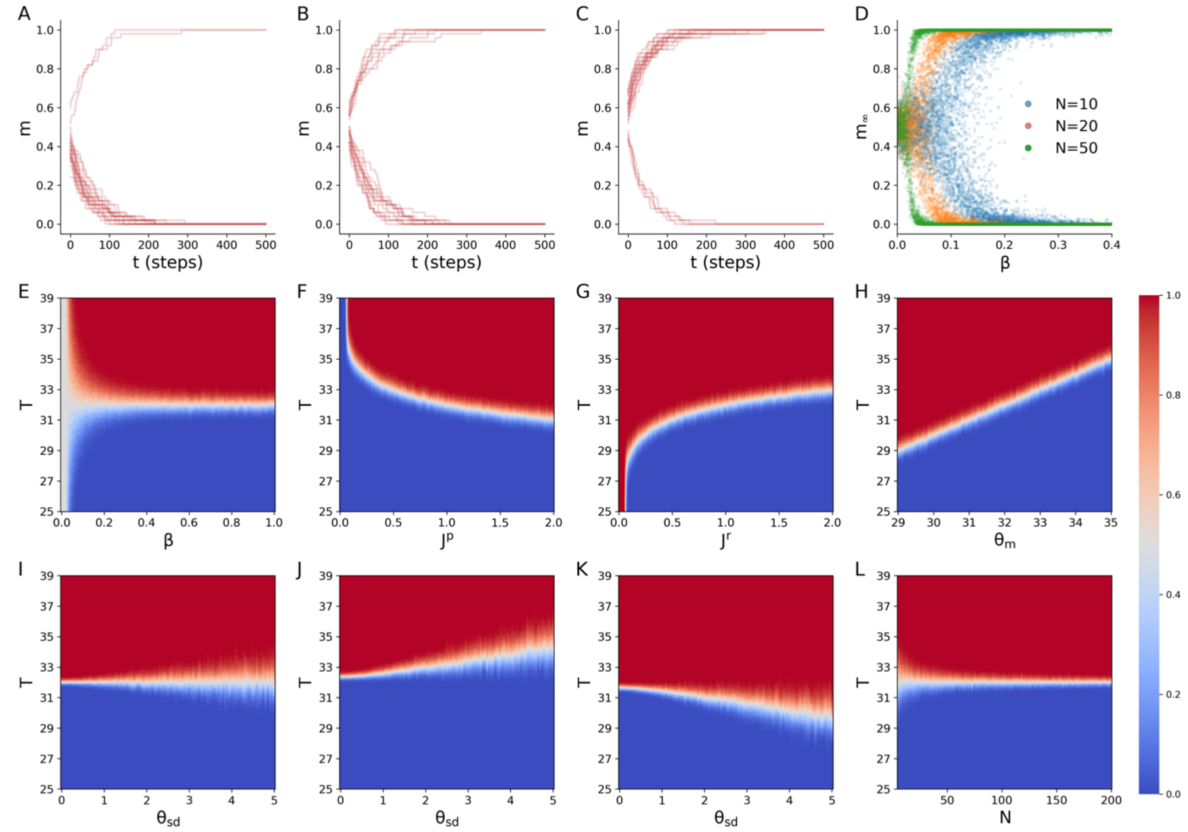

Figure S8: Exploration of the basic model. Unless otherwise stated, all simulation runs are performed for a duration of 20 update cycles. Each cycle consists of N asynchronous updates, with N being the number of ants in the colony. Default parameter settings are N= $50, \beta = 1, J^p = J^r = 1, \theta_m = 32, \theta_{sd} = 2$. For all heatmaps, each pixel represents the average collective response across 100 independent runs of the model. (**A-C**) Evolution of the colony state m as a function of time (measured in cycles), for temperature perturbations of 31.5 (A), 32 (B) and 32.5 (C). For each condition, 25 runs are shown. For threshold temperature perturbations (B), the probability of a colony to respond with a collective nest evacuation is 50%, and the outcome in each case depends on the specific values of individual thresholds of the ants, as well as on the thermal noise. For temperatures below (A) or above (C) the threshold, the probability of a collective response is smaller or larger than 50%, respectively. (**D**) The collective threshold (i.e., the existence of two fixed points for high and low m with a separatrix between them) is contingent on sufficiently high β (i.e., high determinism or low thermal noise). The plot shows the final m for β in the range 0-0.4, with 25 runs of each value of β, for 3 colony sizes. Smaller colonies are more sensitive to thermal noise, and require a larger value of β in order to have a collective threshold. (**E**) The value of the collective threshold does not depend on the thermal noise. The plot shows a heatmap of the collective response probability as a function of temperature and β. Subsequent simulations are therefore done at a high β so that thermal noise is negligible for all colony sizes. (**F-G**) The effect of the interaction parameters J^p (F) and J^r (G) on the collective threshold. As predicted from equation 6, the threshold decreases with J^p and increases with J^r. (**H**) The collective threshold directly corresponds to the individual threshold average θ_m. When $J^p = J^r$, the collective threshold equals θ_m. (**I-K**) The effect of the individual threshold variability θ_{sd} on the collective threshold is contingent on the ratio of J^p and J^r. If $J^p = J^r$ (I), there is no effect on the collective threshold. If $J^p < J^r$ (J), then $m_{th} > 0.5$, and as the distribution of individual thresholds gets wider, the collective threshold is increasing. If $J^p > J^r$ (K), then $m_{th} < 0.5$, and as the distribution of individual thresholds gets wider, the collective threshold is decreasing. (**L**) The collective threshold in the basic model does not depend on colony size N. This holds regardless of the value of the other parameters in the model.

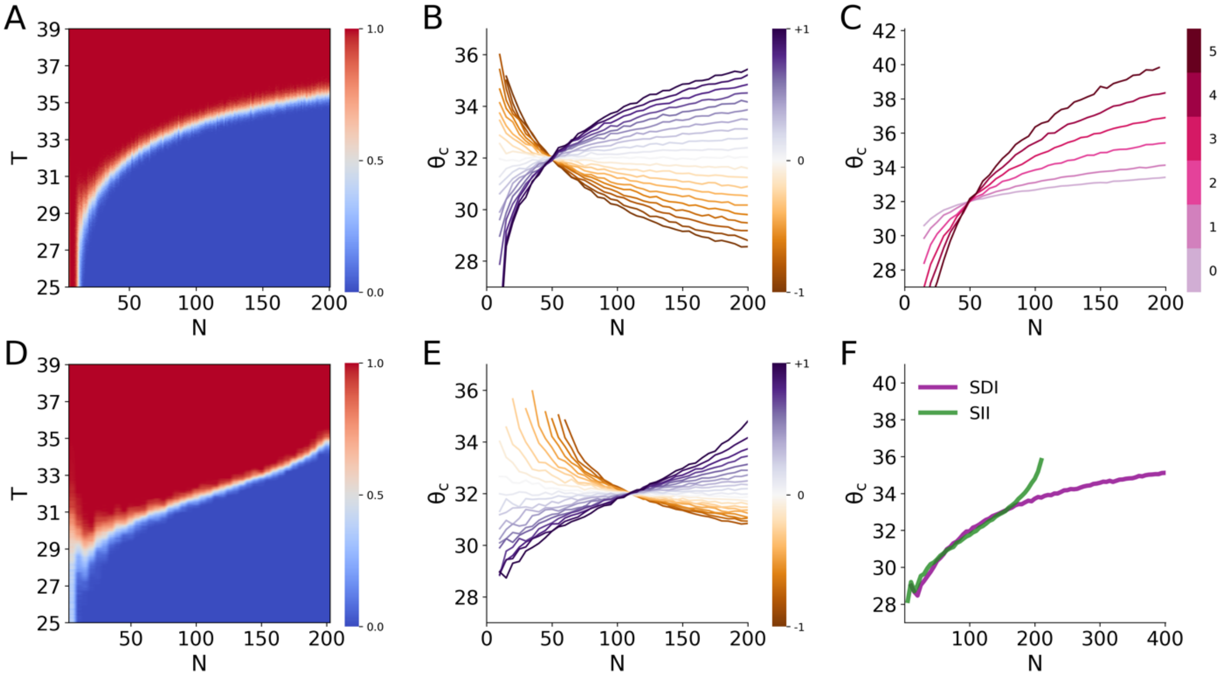

Figure S9: Model with interaction asymmetry. Unless otherwise stated, all simulation runs are performed for a duration of 20 update cycles. Each cycle consists of N asynchronous updates, with N being the number of ants in the colony. Default parameter settings are N= 50, $\beta = 1$,$J^p = J^r = 1, \theta_m = 32, \theta_{sd} = 2, \alpha_r = 1, \alpha_p = 0$. For heatmaps, each pixel represents the average collective response across 100 independent runs of the model. (**A**) Heatmap of the collective response probability as a function of temperature and group size, as in Figure S8L, but with the asymmetric interaction of Equation 8. (**B**) The collective threshold as a function of group size for different value combinations of the scaling parameters α_r and α_p. For each curve, the parameter values were set to $\alpha_r = 1 + \alpha$ and to $\alpha_p = 1 - \alpha$, where the value of α is ranging from -1 (decreasing threshold, brown) to 1 (increasing threshold, purple). The interaction parameters J^p and J^r were scaled so the inhibitory and excitatory components are equal for $N = 50$, resulting in a collective threshold that equals the mean individual threshold. (**C**) The collective threshold as a function of group size for $\alpha_r = 1$, $\alpha_p = 0$, but with different values for θ_{sd}, showing that the variability in individual thresholds determines the magnitude of change in the collective threshold with group size. (**D**) Heatmap of the collective response probability as a function of temperature and group size, as in **A**, for the state-independent inhibition model (Equation 11). (**E**) The collective threshold as a function of group size for the state-independent inhibition model (Equation 11) for different value combinations of the scaling parameters α_r and α_p (values are related, and curves are color coded as in **B**). (**F**) The state-dependent and state-independent inhibition models extrapolate differently to larger colony sizes, giving different predictions. For the state-dependent inhibition (SDI, purple), the collective threshold increase slows down as colony size increases. This is because no matter how strong the inhibition, once the temperature is above the highest individual threshold in the colony, it zeroes out after the first stage of the dynamics. For the state-independent inhibition (SII, green), the collective threshold increase accelerates as colony size increases, until at a critical colony size the colony becomes unresponsive regardless of the temperature value. This is because the inhibition becomes so strong that even if all individuals are perturbed in the first stage, this is still not sufficient to overcome the inhibition, and the ants eventually relax back. The interaction parameters J^p and J^r were scaled so the inhibitory and excitatory components are equal for $N = 100$, resulting in a collective threshold at that group size that equals the mean individual threshold.

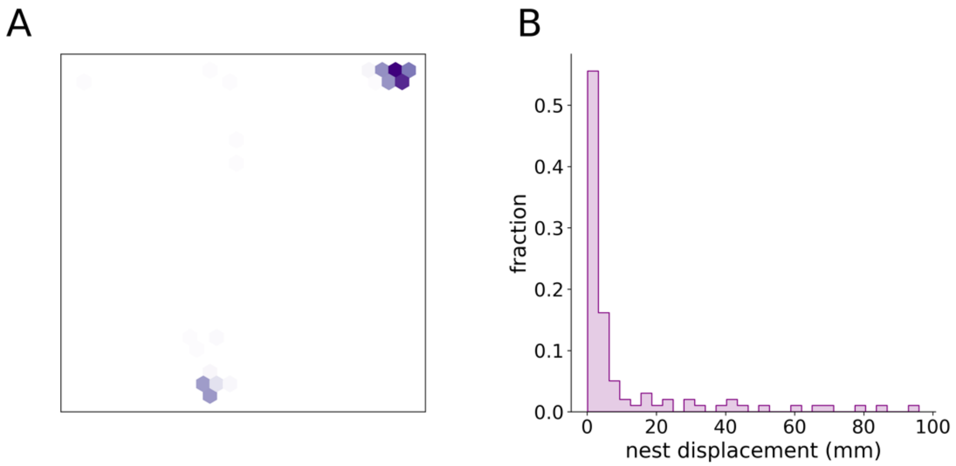

Figure S10: Ants have a tendency to settle at the previous nest site after a perturbation. (**A**) Heatmap depicting the nest locations of one colony of 36 ants throughout a full experiment with variable perturbation amplitudes (>48hrs). (**B**) Histogram of nest displacement distances following full evacuations of the nest in response to temperature perturbations. Data points are pooled across all colonies from the experiment with 36 individually tagged ants per colony and variable perturbation amplitudes. The plot shows a high tendency of colonies to return to their pre-perturbation nest site. This could be explained by a hitherto unknown, weakly volatile pheromone that marks the nest location.

Movie S1: Evacuation response to a strong temperature perturbation. The video shows a typical nest evacuation of a colony of 36 clonal raider ants in response to a strong (40°C) step increase in temperature. For visualization purposes, the video is background subtracted to show only the behavior of the ants and is sped up x3. The time counter shows the time in seconds relative to the onset of the perturbation.

Movie S2: Aborted response to a mild temperature perturbation. The video shows an example of an aborted response of a colony of 36 clonal raider ants in response to a mild (33°C) step increase in temperature. For visualization purposes, the video is background subtracted to show only the behavior of the ants and is sped up x3. The time counter shows the time in seconds relative to the onset of the perturbation.
